# Immature teratoma arising from uterine corpus in an 11‐year‐old girl: Case report and review of the literature

**DOI:** 10.1111/jog.14547

**Published:** 2020-11-16

**Authors:** Yinling Zhao, Tianhui Xu, Xinhua Bu, Donglan Yuan, Yiqun Wu, Hua Qian

**Affiliations:** ^1^ Department of Obstetrics and Gynaecology Hospital Affiliated 5 to Nantong University (Taizhou People's Hospital) Taizhou China

**Keywords:** immature teratoma, mature teratoma, uterine teratoma

## Abstract

Teratomas are one of the most common germ cell tumors, and they usually occur in ovaries. Extragonadal teratomas are rare, especially immature ones. Only several cases of primary teratomas of the uterus have been reported since 1929. Here, the case of an 11‐year‐old patient who had a 6‐month history of sustained abnormal vaginal discharge is presented. Transabdominal ultrasonography revealed a solid mass in her uterus, resulting in the patient undergoing surgery. Examination of PET‐CT scans revealed a mass in the right ovary of the patient 20 days after surgery. The patient underwent a second surgery followed by chemotherapy. This is the youngest case among reported patients of primary immature uterine teratoma, and this patient showed no evidence of recurrence during 2 years of follow‐up.

## Introduction

Teratomas are one of the most common germ cell tumors classifiable as mature or immature.^1^ The tumor characteristics and the extent of malignancy depend on the differentiation degree. An immature teratoma is a malignant tumor, which accounts for 1–3% of ovarian teratomas.^2^ Teratomas usually arise in the gonads, while extragonadal teratomas are very rare and account for 2–5% of germ cell tumors.^3^ Primary teratomas of the uterus have rarely been reported since 1929.^4^, ^5^, ^6^, ^7^ Here, the case of an 11‐year‐old patient who had an immature teratoma in her uterus and a mature teratoma in her ovary is presented.

## Case Report

**Figure 1:**
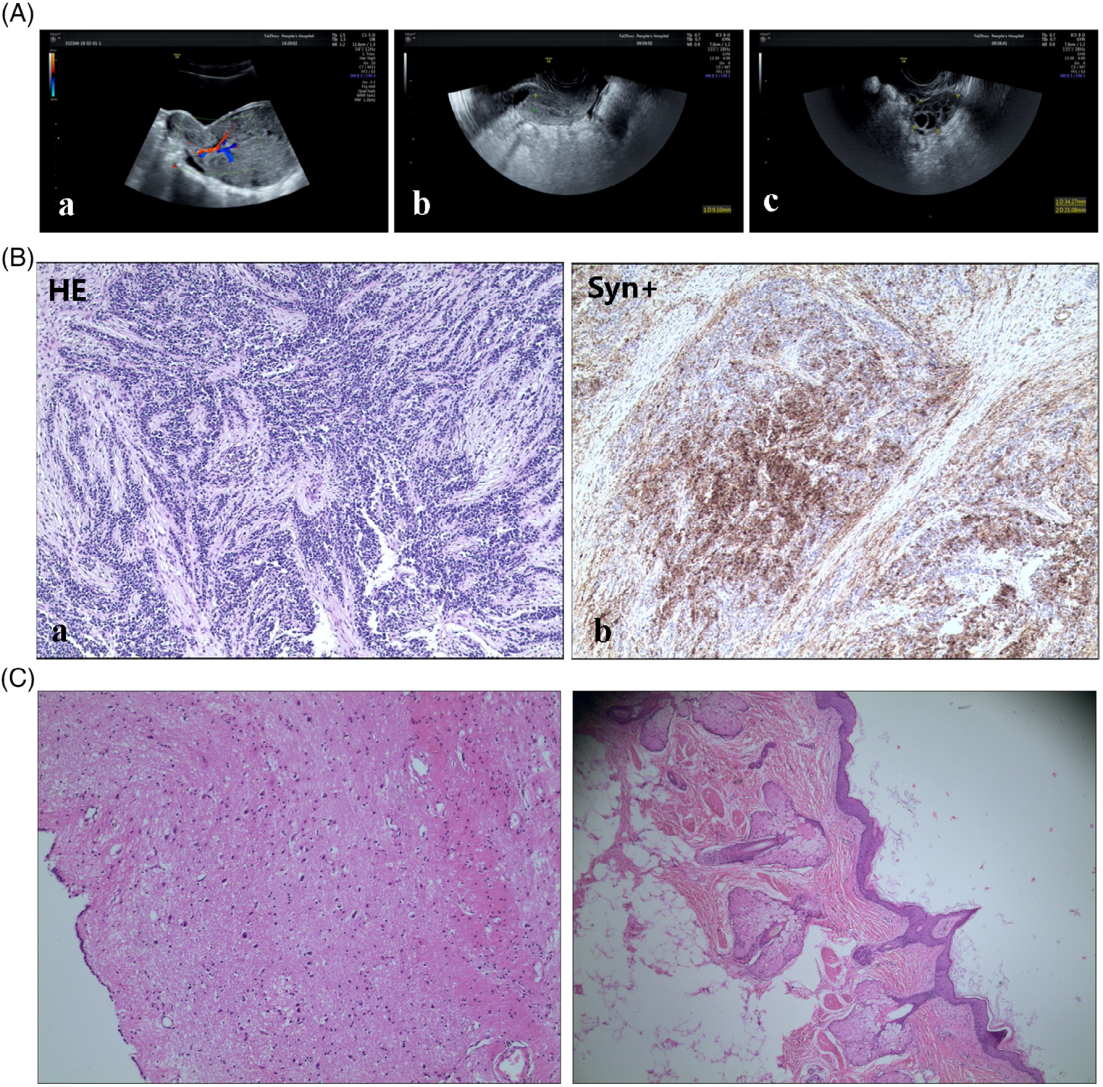
A (a) Ultrasonography showing a solid mass measuring 11 × 6.5 cm from uterus to vagina with heterogenous internal echo; (b) ultrasound image after lesion excision; (c) ultrasonography showing a solid mass of the right ovary measuring 3.1 × 3.2 cm. B (a) Histopathological appearances of malignant uterine teratoma; (b) immunohistochemical analysis showing positive staining for Syn. C Histopathological appearances of mature ovarian teratoma.

An 11‐year‐old girl with no special medical history went to the hospital in February 2018 with a 6‐month history of sustained abnormal vaginal discharge. A mass could be seen in the orifice of her vagina through a gynecologic examination. Transabdominal ultrasonography revealed a solid mass measuring 11 × 6.5 cm from the uterus to the vagina with heterogeneous internal echo (Figure 1A). Tumor markers, including AFP, CA125 and CA19‐9, were all within normal limits. The first menstrual period of the patient was in July 2017. The menstrual cycle was not regular with about 30–50 days. The last menstruation was on December 20, 2017. The fertility of the patient was chosen to be retained, considering that she is an 11‐year‐old girl. The mass was moved by a hysteroscopic transcervical resection in February 2018. During surgery, a 12 × 6 cm mass was found protruding from the posterior uterine wall into the vagina through the cervical canal. The characteristics of the mass were yellow and soft. Most of the mass was then cut out by an experienced gynecologist. Microscopically, the mass was revealed to be an immature teratoma composed of mature and immature embryonic layers, especially including immature neuroepithelial elements. Subsequent Immunohistochemistry showed positive staining of Syn+, Nestin+, CD99+, Fli‐1+, SAL4+, P53+, AEI/AE3− and Desmin− (Figure 1B).

Examination of ultrasound and CT scans showed no residual teratoma after the operation, but a mass was present in the right ovary of the patient 20 days after surgery. It measured about 3.1 × 3.2 cm in size, with fat density and dot‐strip calcification, suggesting that it may be a teratoma. The mass was not noticed before. Inevitably, the patient underwent a second operation via a laparoscope in March 2018. During the operation, a 3.1 × 3.2 cm mass was removed from the right ovary. Microscopically, the mass was revealed to be mature teratoma (Figure 1C). The following treatment strategy included six courses of chemotherapy (BEP regimen) based on bleomycin (15 mg/body, days 1–2), etoposide (100 mg/m^2^, days 1–2) and cisplatin (30 mg/m^2^, days 1–2) every 3 weeks. The patient was well after 2 years of follow‐up post‐surgery. No residual tumors were found through regular examinations of ultrasound and CT scans. As well, the patient no longer has any abnormal symptoms.

## Discussion

Extragonadal germ cell tumors can arise anywhere along germ cell migration routes.^2^ Teratomas are the most common germ cell tumors, and the occurrence of primary uterine teratomas is rare. The first uterine teratoma case was reported in 1929, with only a few cases being reported after that. Primary immature uterine teratoma was particularly rare, with only eight cases reported according to statistics investigated for this paper (Table 1). However, this case is the first case where the patient was only 11 years old, who was also the youngest case among all reported patients of primary immature uterine teratoma.

**Table 1:** Case reports of uterine immature teratoma in English literature

Symptoms	Age	Site of tumor	Histology	Treatment	Follow‐up	Author
Vaginal bleeding	37	Not specified	Immature teratoma, grade2+ endometrioid carcinoma	TAH+radiotherapy	No recurrence	Ansah‐Boateng *et al*. (1985)
Pelvic pain, lower abdominal distention	36	fundus	Immature teratoma, grade 3	TAH+chemotherapy (VAC*2)	No recurrence after 5 years	Iwanaga *et al*. (1993)
Vaginal bleeding Pelvic pain	15	Not specified	Immature teratoma, high grade	Lesion excision+chemotherapy	Not specified	Gomez‐Lobo *et al*. (2007)
Vaginal bleeding	82	Corpus	Immature teratoma	TAH‐BSO	Relapse after 6 months	Newsom‐Davis *et al*. (2009)
Urinary symptoms, lower abdominal distention	56	Corpus	Immature teratoma	TAH	Relapse After 3 months	Ben‐Ameur *et al*. (2013)
vaginal bleeding, malodorous discharge	23	Corpus and cervix	Immature teratoma, grade 2+ carcinoid elements	TAH + BSO + lymph node Dissection+BEP*3	Disease‐free after 12 months	Teixeira Souza *et al*. (2014)
menometrorrhagia and intermenstural bleeding	29	cervix	Immature teratoma, grade 3	TAH+BSO+lymph node Dissection+omentectomy	Disease‐free after 36 months	Hana Saffar *et al*. (2016)
Vaginal bleeding	46	endometrium	Immature teratoma, grade 1	Lesion excision	Disease‐free after 5 months	Simona Stolnicu *et al*. (2017)
Abnormal vaginal discharge	11	Corpus	Immature teratoma	Lesion excision+BEP	Disease‐free after 2 years	Our case, 2018

BEP: bleomycin, etoposide, cisplatin; BSO: bilateral salpingooophorectomy; EP: etoposide, cisplatin; ET: etoposide, paclitaxel; IT: immature teratoma; ns: not specified; TAH: total abdominal hysterectomy; TP: palcitaxel, cisplatin; VAC: vincristine, actinomycin‐D, and cyclophosphamide.

Two main theories about uterine teratoma origins can be discussed. The first theory suggests that they arise from primordial fetal germ cells when migrating abnormally from the fetal yolk sac endoderm to the gonadal ridge during early embryogenesis. These tumors are considered to arise in middle structures like the uterus.^8^ Another theory proposes that they arise from residual fetal tissue implemented during missed abortion or fertility processes.^9^ The patient in the case of this paper is an 11‐year‐old girl with neither a history of abortion nor instrumentation. Therefore, the second theory was ruled out in this case.

In uterine teratoma reports, patient ages ranged from 15 to 90. In this case, the age of the patient, 11‐year‐old, was the youngest in all reports since 1992. Usually, no special symptoms occur with uterine teratoma, including vaginal bleeding, lower abdominal pain, pelvic inflammation and other single or combined symptoms.^10^ In this case, the vaginal fluid was treated for 6 months. For the uterine teratoma diagnosis, auxiliary examination mainly includes ultrasound, CT scan or magnetic resonance imaging. Imaging manifestations are also varied without specificity, and the examination results will be affected by location, size and so on. Therefore, making an accurate diagnosis via auxiliary examination before the operation is difficult, and it also needs to be differentiated from other myomas.^11^ The final pathology is the gold standard for disease diagnosis. In this case, the initial gynecological examination of the patient found a vaginal mass exfoliation, followed by ultrasonography. Before the operation, whether or not it was a uterine teratoma was unclear, but this was not determined until intraoperative and postoperative pathology revealed it to be an immature uterine teratoma. The main treatment of immature uterine teratoma is surgery, consisting of a complete tumor excision or radical hysterectomy with or without pelvic and para‐aortic lymphadenectomy.^2^, ^10^ Considering the age of the patient, the operative method was chosen to preserve the fertility of the patient. Postoperative PET‐CT scan reexamination revealed a mature teratoma of the right ovary, which had not been noticed before, and the reason for it was unknown. Therefore, a second operation was unavoidable.

No uniform consensus exists regarding chemotherapy and radiotherapy of uterine immature teratomas.^12^ Among several reported cases, various therapeutic methods have been used; however, uterine teratoma prognosis is uncertain because of the small number of reported cases. The BEP regiment was chosen in this case, considering the operation mode and the postoperative state of the patient.^13^ The patient showed no evidence of recurrence during 2 years of resulting follow‐up. In conclusion, the therapeutic standard and prognosis of immature uterine teratoma remain unknown.

## Disclosure

None declared.
